# Association of the American Heart Association’s new “Life’s Essential 8” with all-cause and cardiovascular disease-specific mortality: prospective cohort study

**DOI:** 10.1186/s12916-023-02824-8

**Published:** 2023-03-29

**Authors:** Jiahong Sun, Yanzhi Li, Min Zhao, Xiao Yu, Cheng Zhang, Costan G. Magnussen, Bo Xi

**Affiliations:** 1 Department of Epidemiology/Shandong Provincial Clinical Research Center for Emergency and Critical Care Medicine, School of Public Health/Qilu Hospital, Cheeloo College of Medicine, Shandong University, 44 Wen Hua Xi Road, Jinan, 250012 China; 2 Department of Medical Statistics and Epidemiology, School of Public Health, Sun Yat-Sen University, Guangzhou, China; 3 Department of Nutrition and Food Hygiene, School of Public Health, Cheeloo College of Medicine, Shandong University, Jinan, China; 4 Key Laboratory Experimental Teratology of the Ministry of Education, Department of Physiology, School of Basic Medical Sciences, Cheeloo College of Medicine, Shandong University, Jinan, China; 5 Key Laboratory of Cardiovascular Remodeling and Function Research, Chinese Ministry of Education, Chinese National Health Commission and Chinese Academy of Medical Sciences, The State and Shandong Province Joint Key Laboratory of Translational Cardiovascular Medicine, Department of Cardiology, Qilu Hospital, Cheeloo College of Medicine, Shandong University, Jinan, China; 6 Baker Heart and Diabetes Institute, Melbourne, VIC Australia; 7 Research Center of Applied and Preventive Cardiovascular Medicine, University of Turku, Turku, Finland; 8 Centre for Population Health Research, University of Turku and Turku University Hospital, Turku, Finland

**Keywords:** Cardiovascular health, Life’s Essential 8, Cardiovascular disease, Mortality, Prospective study

## Abstract

**Background:**

The American Heart Association recently updated its construct of what constitutes cardiovascular health (CVH), called *Life’s Essential 8*. We examined the association of total and individual CVH metrics according to *Life’s Essential 8* with all-cause and cardiovascular disease (CVD)-specific mortality later in  life.

**Methods:**

Data were from the National Health and Nutrition Examination Survey (NHANES) 2005–2018 at baseline linked to the 2019 National Death Index records. Total and individual CVH metric scores including diet, physical activity, nicotine exposure, sleep health, body mass index, blood lipids, blood glucose, and blood pressure were classified as 0–49 (low level), 50–74 (intermediate level), and 75–100 (high level) points. The total CVH metric score (the average of the 8 metrics) as a continuous variable was also used for dose–response analysis. The main outcomes included all-cause and CVD-specific mortality.

**Results:**

A total of 19,951 US adults aged 30–79 years were included in this study. Only 19.5% of adults achieved a high total CVH score, whereas 24.1% had a low score. During a median follow-up of 7.6 years, compared with adults with a low total CVH score, those with an intermediate or high total CVH score had 40% (adjusted hazard ratio [HR] 0.60, 95% confidence interval [CI] 0.51–0.71) and 58% (adjusted HR 0.42, 95% CI 0.32–0.56) reduced risk of all-cause mortality. The corresponding adjusted HRs (95%CIs) were 0.62 (0.46–0.83) and 0.36 (0.21–0.59) for CVD-specific mortality. The population-attributable fractions for high (score ≥ 75 points) vs. low or intermediate (score < 75 points) CVH scores were 33.4% for all-cause mortality and 42.9% for CVD-specific mortality. Among all 8 individual CVH metrics, physical activity, nicotine exposure, and diet accounted for a large proportion of the population-attributable risks for all-cause mortality, whereas physical activity, blood pressure, and blood glucose accounted for a large proportion of CVD-specific mortality. There were approximately linear dose–response associations of total CVH score (as a continuous variable) with all-cause and CVD-specific mortality.

**Conclusions:**

Achieving a higher CVH score according to the new *Life’s Essential 8* was associated with a reduced risk of all-cause and CVD-specific mortality. Public health and healthcare efforts targeting the promotion of higher CVH scores could provide considerable benefits to reduce the mortality burden later in life.

**Supplementary Information:**

The online version contains supplementary material available at 10.1186/s12916-023-02824-8.

## Background

Cardiovascular disease (CVD) is a leading cause of mortality worldwide [1]. The economic burden of CVD and related mortality in the United States (US) is particularly troubling with an estimated average annual cost of US $363.4 billion from 2016 to 2017 [2, 3]. Obesity, physical inactivity, poor diet, smoking, insufficient sleep duration, high blood pressure, diabetes, and dyslipidemia among adults have been identified as major risk factors to address for the successful prevention of CVD and related mortality [4].

In 2010, The American Heart Association (AHA) established *Life’s Simple 7* (ideal goals for 7 behavioral and health factors of healthy diet, physical activity, normal body mass index (BMI), no smoking, normal blood pressure, normal fasting glucose, and normal total cholesterol) to promote ideal cardiovascular health (CVH) [5]. Although the implementation of interventions targeting these factors has obtained favorable effects on CVH promotion, the prevalence of ideal CVH is extremely low (i.e., < 1%) among the US population [6]. Meta-analyses from cohort studies have shown the association of achieving a greater number of ideal cardiovascular metrics with a reduced risk of CVD and all-cause mortality (risk ratio (RR) [95% CI] of the highest vs. the lowest category: 0.22 [0.11–0.42] for CVD, and 0.54 [0.41–0.69] for all-cause mortality) [7, 8]. However, some included studies were solely adjusted for sex and age and failed to adjust for other important risk factors such as education level and family income [8]. In addition, *Life’s Simple 7* has several limitations including the confined scope of health behaviors (e.g., not including sleep health), a crude additive scoring, and less sensitivity to interindividual differences and intraindividual change [9].

To overcome the limitations of *Life’s Simple 7*, the AHA’s Strategic Planning Task Force and Statistical Committee recently recommended *Life’s Essential 8* goals to maintain optimal CVH, which added sleep health, and updated and refined the other 7 components that were part of *Life’s Simple 7* (particularly, changes to the scoring algorithm of components in *Life’s Essential 8*) [9]. To our knowledge, no studies have assessed the performance of these new CVH metrics in *Life’s Essential 8* in relation to mortality. From a public health perspective, it is important to validate the implementation and utility of these new CVH metrics in *Life’s Essential 8* to promote awareness and adherence to the current recommendations, thereby reducing the burden of CVD and related mortality.

Therefore, based on nationally representative data and using the new scoring algorithm, we examined the association of the overall and individual CVH metric scores defined in *Life’s Essential 8* with all-cause and CVD-specific mortality among US adults. In addition, we also calculated population-attributable fractions (PAFs) of these new CVH metrics scores in relation to mortality risk.

## Methods

### Study population

Data from the National Health and Nutrition Examination Survey (NHANES) conducted during the period of 2005–2018 were used for this study. The NHANES is an ongoing, national, cross-sectional survey with a complex, stratified, and multistage probability sampling design of the noninstitutionalized US civilian population. Information on the introduction, content, and operations of the NHANES is available elsewhere [10]. The NHANES was approved by the National Center for Health Statistics Research Ethics Review Board, and written informed consent was obtained from all participants.

As sleep health information was first collected in the 2005–2006 NHANES, we used NHANES data collected from 2005 to 2018 as baseline data. A total of 39,749 adults aged ≥ 20 years participated in the 2005 to 2018 NHANES, of which 4979, 5935, 6218, 5560, 5769, 5719, and 5569 were in the survey cycles of 2005–2006, 2007–2008, 2009–2010, 2011–2012, 2013–2014, 2015–2016, and 2017–2018, respectively. After excluding participants with the age of < 30 or > 79 years old (*n* = 9534), those with missing data on cardiovascular metrics (*n* = 8208) or potential covariates (*n* = 1747), those who were pregnant or breastfeeding (*n* = 288), and those with ineligible data on death or follow-up years (*n* = 21), a total of 19,951 adults aged 30–79 years were included in this study. The basic characteristics between follow-up participants and those without CVH metrics are shown in Additional file 1: Table S1. The detailed flow chart is shown in Additional file 1: Fig. S1.

### Definitions of updated cardiovascular metrics in *Life’s Essential 8*

#### Diet

Dietary information in this study was obtained using a self-reported food frequency questionnaire. Participants with 2 days of dietary information were included for data analysis (those with 1-day data only were excluded). The AHA proposed a new method for assessing dietary quality based on quantities of adherence to the Healthy Eating Index 2015 at a population level (1st–24th, 25th–49th, 50th–74th, 75th–94th, and ≥ 95th percentile values, corresponding to 0, 25, 50, 80, and 100 points, respectively) [9].

#### Physical activity

Physical activity was assessed based on self-reported minutes of moderate or vigorous physical activity per week collected by a questionnaire. Physical activity time was classified as 0, 1–29, 30–59, 60–89, 90–119, 120–149, and ≥ 150 min per week, corresponding to 0, 20, 40, 60, 80, 90, and 100 points, respectively [9].

#### Nicotine exposure

Information on self-reported nicotine exposure collected in the questionnaire was used. In addition to combustible cigarette use, the AHA added the use of other inhaled nicotine delivery systems such as vaping devices, e-cigarettes, and secondhand tobacco smoke to the definition in *Life’s Essential 8*. There are 5 nicotine exposure categories including current smoker, former smoker (quit < 1 year) or currently using inhaled nicotine delivery systems, former smoker (quit 1 to < 5 years), former smoker (quit ≥ 5 years), and never smoker, corresponding to 0, 25, 50, 80, and 100 points, respectively). Twenty points were subtracted for adults living with active indoor smokers at home [9].

#### Sleep health

Sleep health was a new metric in *Life’s Essential 8* goals based on the self-reported average hours of sleep per night collected by a questionnaire. Sleep hours per night were classified as < 4, 4 to < 5, 5 to < 6 or ≥ 10, 6 to < 7, 9 to < 10, and 7 to < 9 h, corresponding to 0, 20, 40, 70, 90, and 100 points, respectively [9].

#### Body mass index

Objectively measured weight and height were used to calculate BMI (i.e., weight in kilograms divided by height in meters squared). BMI levels were classified as ≥ 40.0, 35.0–39.9, 30.0–34.9, 25.0–29.9, and < 25.0 kg/m^2^, corresponding to 0, 15, 30, 70, and 100 points, respectively [9].

#### Blood lipids

Blood samples were used to measure the total and high-density lipoprotein (HDL) cholesterol. Non-HDL cholesterol was calculated as total cholesterol subtracting HDL cholesterol. Non-HDL cholesterol levels were classified as ≥ 220, 190–219, 160–189, 130–159, and < 130 mg/dL, corresponding to 0, 20, 40, 60, and 100 points, respectively [9].

#### Blood glucose

Fasting blood samples were used to measure fasting blood glucose (FBG). Both fasting and non-fasting blood samples were used to measure HbA1c. Blood glucose levels were classified as diabetes with HbA1c ≥ 10.0%, diabetes with HbA1c of 9.0–9.9%, diabetes with HbA1c of 8.0–8.9%, diabetes with HbA1c of 7.0–7.9%, diabetes with HbA1c < 7.0%, no diabetes and FBG of 100–125 mg/dL or HbA1c of 5.7–6.4%, and no history of diabetes and FBG < 100 mg/dL or HbA1c < 5.7%, corresponding to 0, 10, 20, 30, 40, 60, and 100 points, respectively [9].

#### Blood pressure

Blood pressure was measured using an appropriately sized cuff. Blood pressure levels were classified as systolic blood pressure ≥ 160 mmHg or diastolic blood pressure ≥ 100 mmHg, 140–159 or 90–99 mmHg, 130–139 or 80–89 mmHg, 120–129/ < 80 mmHg, and < 120/80 mmHg, corresponding to 0, 25, 50, 75, and 100, respectively. Twenty points were subtracted if the blood pressure levels were treated [9].

We calculated the total score using the average of all 8 individual cardiovascular metric scores according to AHA recommendation [9].

### Definition of mortality

Baseline data from NHANES 2005–2018 were linked to mortality data from the National Death Index death certificate records until December 31, 2019, matched using a probabilistic matching algorithm to identify mortality status [11]. The outcomes included all-cause mortality and CVD-specific mortality (codes I00–I09, I11, I13, I20–I51, and I60–I69) using the International Classification of Disease Tenth Revision.

### Study covariates

Study covariates included sex (male and female), age group (30–49, 50–64, and 65–79 years), race/ethnicity (Hispanic, non-Hispanic White, non-Hispanic Black, and others), educational level (< high school, high school, some college or associates degree, and college graduate or above), marital status (married, divorced/separated/widowed, and unmarried/cohabitation), and the ratio of family income to poverty (< 1.3, 1.3–2.99, and ≥ 3.0).

### Statistical analysis

We calculated the mean scores and 95% confidence intervals (CIs) and the mean levels and 95% CIs of each CVH metric by total CVH scores of 0–49 (low), 50–74 (intermediate), and 75–100 points (high), which were determined according to the subsequent dose–response relationship between CVH score and mortality. We also estimated the proportions (%) of sex, age group, race/ethnicity, education level, marital status, and the ratio of family income to poverty by low, intermediate, and high levels of total CVH scores. A direct method of standardization was used to calculate the age- and sex-standardized rates of all-cause and CVD-specific mortality per 1000 person-years (the number of follow-up person-years estimated from baseline to the end of the study, loss to follow-up, death, or December 31, 2019, whichever came first) according to the three categories of the total score of CVH metrics (low, intermediate, high). Kaplan–Meier survival curves were generated for the calculation of cumulative mortality using three score categories of CVH metrics (low, intermediate, high), compared using the log-rank test. Competing risk analysis was used to estimate the cumulative incidence of CVD-specific mortality in consideration of non-CVD deaths as the competing events. We used Cox proportional hazards regression models to examine the associations of combined and single CVH metric scores (low level as the reference) with all-cause and CVD-specific mortality with adjustment for sex, age, race/ethnicity (model 1), and full covariates (model 2) (i.e., sex, age, race/ethnicity, education level, marital status, ratio of family income poverty, diet, nicotine exposure, physical activity, sleep health, BMI, blood glucose, blood lipids, blood pressure, and history of heart disease and stroke). When the association between each CVH metric and mortality was evaluated, this metric was excluded from adjustment. The adjusted PAFs of high (score ≥ 75 points) vs. intermediate or low CVH score (score < 75 points) were estimated for the proportion of all-cause and CVD-specific mortality that could be avoided if participants with low or intermediate CVH scores achieve high CVH scores [12]. Furthermore, the adjusted PAFs of high (score ≥ 85 points) vs. intermediate or low CVH score (score < 85 points) were also estimated since the choice of score ≥ 75 points used for the “high score” definition in our main analyses was somewhat arbitrary. Sensitivity analyses were performed to examine the association of total CVH metric score with all-cause and CVD-specific mortality stratified by sex, age group, race/ethnicity, educational level, marital status, and ratio of family income to poverty. Another sensitivity analysis was conducted with the exclusion of adults with a history of CVD or death within the first 2 years of follow-up to assess whether the findings were influenced by reverse causation. Restricted cubic spline models with three knots (10th, 50th, and 90th percentiles) were conducted to estimate the dose–response association of total CVH metric score with all-cause and CVD-specific mortality, with 50 points of CVH score as the reference. We used appropriate sample weights, as well as strata and primary sampling units to obtain nationally representative estimates of the US population. All analyses were performed using STATA version 16.0 (Stata Corporation, College Station, TX, USA). A two-sided *P* < 0.05 was considered statistically significant.

## Results

The characteristics of participants across the three categories of total CVH score (low, intermediate, high) are shown in Table 1. Only 19.5% of adults achieved a high total CVH score, whereas 24.1% had a low score. Adults with higher CVH scores were more likely to be female, younger, non-Hispanic White, and married and have a high education level and high ratio of family income to poverty (all *P* < 0.001). Adults with a history of heart disease or stroke were less likely to have high CVH scores (all *P* < 0.001).

**Table 1:** Characteristics of US adults by three categories of total cardiovascular health (CVH) score, 2005–2018

Characteristic	Total CVH scores			*P* value
	**0–49**	**50–74**	**75–100**	
**Overall**	4810	11,257	3884	
**Sex (%)**				< 0.001
Male	52.3	50.6	39.6	
Female	47.7	49.4	60.4	
**Age group (%)**				< 0.001
30–49 years	34.5	41.4	57.3	
50–64 years	40.1	34.0	25.8	
65–79 years	25.4	24.6	16.9	
**Race/ethnicity (%)**				< 0.001
Hispanic	21.9	26.5	20.2	
Non-Hispanic White	44.7	43.1	51.2	
Non-Hispanic Black	27.9	21.4	11.5	
Others	5.5	9.0	17.0	
**Education level (%)**				< 0.001
< High school graduate	32.2	23.1	10.1	
High school graduate	28.6	23.6	13.0	
Some college or associates degree	29.0	30.8	24.9	
College graduate or above	10.2	22.6	52.1	
**Marital status (%)**				< 0.001
Married	50.0	59.3	69.3	
Divorced/separated/widowed	30.5	23.6	15.6	
Unmarried/cohabitation	19.4	17.1	15.1	
**Ratio of family income to poverty**				< 0.001
< 1.30	41.3	27.4	14.9	
1.30–2.99	33.4	31.8	24.1	
≥ 3.00	25.3	40.9	61.1	
**History of heart disease (%)**				< 0.001
Yes	15.0	7.7	3.5	
No	85.0	92.3	96.6	
**History of stroke (%)**				< 0.001
Yes	7.2	3.3	1.1	
No	92.8	96.7	98.9	
**Diet**				
Mean score (95% CI)	24.8 (23.6–26.0)	41.4 (40.5–42.4)	64.5 (63.0–66.0)	< 0.001
**Physical activity**				
Mean (95% CI), min/week	30.0 (24.4–35.6)	143.7 (135.6–151.8)	291.6 (276.2–307.0)	< 0.001
Mean score (95% CI)	11.1 (9.8–12.4)	46.1 (44.6–47.6)	86.1 (84.7–87.5)	< 0.001
**Nicotine exposure**				
Mean score (95% CI)	40.9 (39.1–42.7)	70.1 (68.9–71.2)	90.1 (89–91.2)	< 0.001
**Sleep health**				
Mean (95% CI), h/day	6.7 (6.6–6.7)	7.1 (7.1–7.2)	7.4 (7.3–7.4)	< 0.001
Mean score (95% CI)	69.7 (68.2–71.2)	84.3 (83.8–84.9)	92.5 (91.9–93.2)	< 0.001
**Body mass index**				
Mean (95% CI), kg/m^2^	34.1 (33.7–34.5)	29.8 (29.6–30.0)	25.3 (25.1–25.5)	< 0.001
Mean score (95% CI)	35.2 (33.7–36.8)	55.4 (54.5–56.4)	81.2 (80.1–82.3)	< 0.001
**Blood lipids (non-HDL cholesterol)**				
Mean (95% CI), mg/dL	165.6 (163.1–168.1)	147.1 (145.7–148.5)	127.3 (125.7–128.9)	< 0.001
Mean score (95% CI)	43.9 (42.2–45.7)	59.2 (58.3–60.0)	76.6 (75.1–78.1)	< 0.001
**Blood glucose**				
Mean score (95% CI)	59.1 (58.0–60.2)	76.3 (75.6–77.0)	90.1 (89.1–91.1)	< 0.001
Fasting glucose, mg/dL, mean (95% CI)	125.1 (122.2–128.0)	109.5 (108.3–110.8)	99.1 (97.8–100.5)	< 0.001
HbA1c, %, mean (95% CI)	6.2 (6.2–6.3)	5.6 (5.6–5.7)	5.3 (5.3–5.3)	< 0.001
**Blood pressure**				
Mean score (95% CI)	44.6 (43.1–46.0)	64.8 (63.9–65.8)	85.7 (84.6–86.8)	< 0.001
Systolic, mmHg, mean (95% CI)	132.4 (131.6–133.2)	123.4 (122.9–123.8)	113.9 (113.4–114.5)	< 0.001
Diastolic, mmHg, mean (95% CI)	75.0 (74.3–75.8)	72.3 (71.9–72.7)	69.3 (68.8–69.9)	< 0.001

*CVD* cardiovascular disease, *CI* confidence interval

**Fig. 1:**
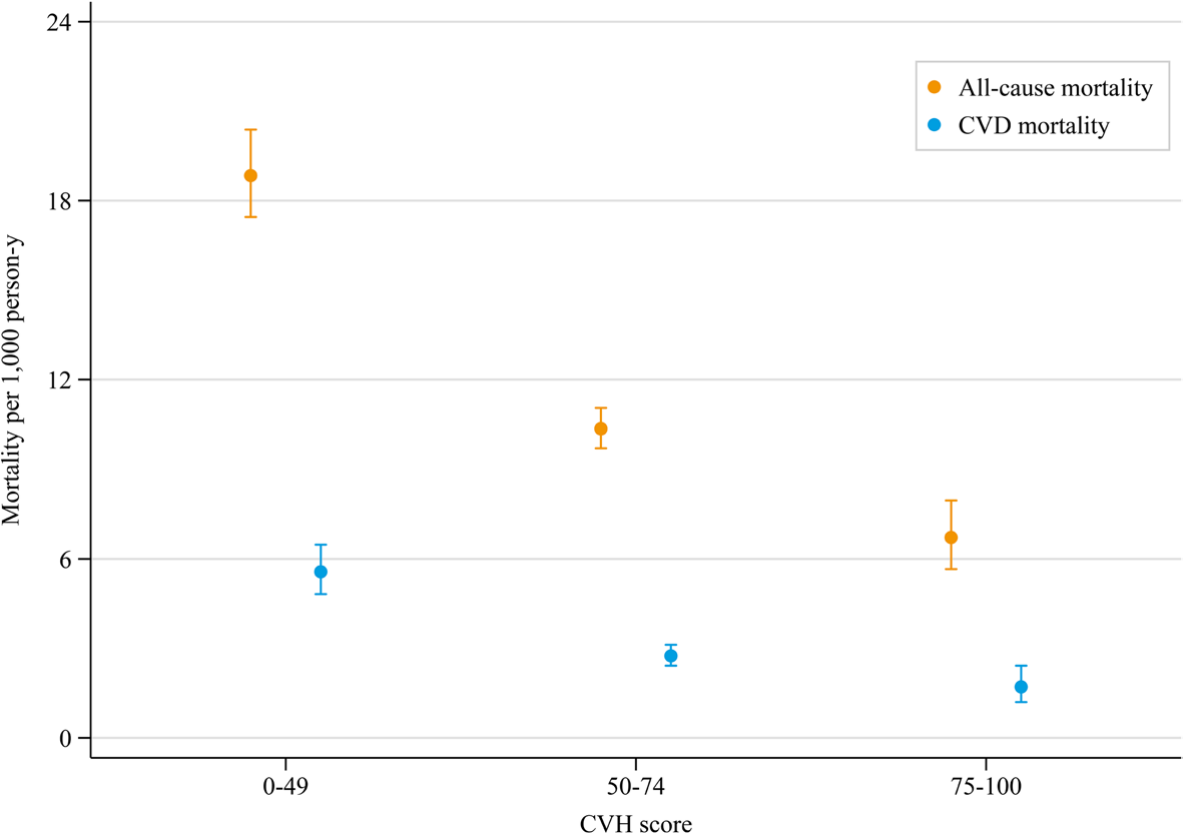
Age- and sex-standardized all-cause and CVD-specific mortality rate per 1000 person-years by total scores of cardiovascular health metrics

**Fig. 2:**
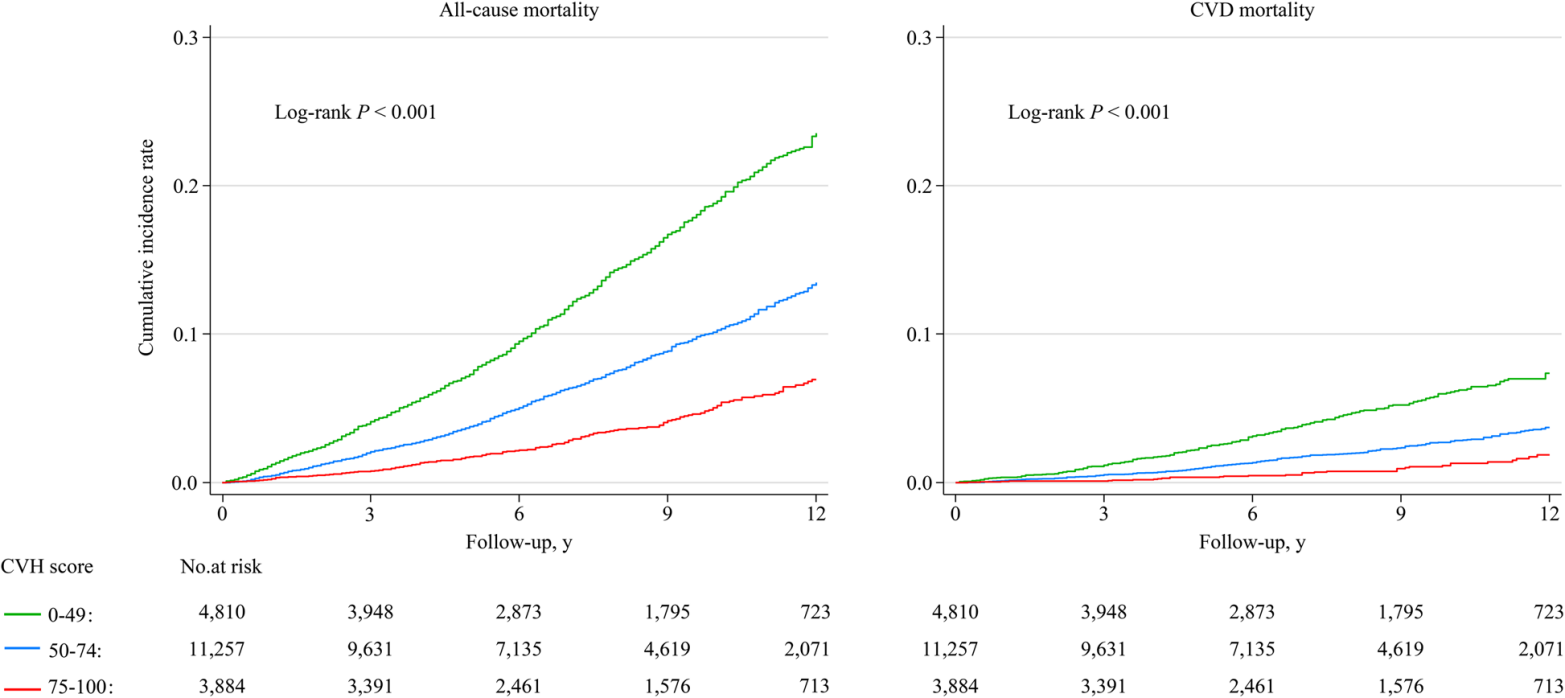
Kaplan–Meier curves for cumulative all-cause and CVD-specific mortality by total scores of cardiovascular health metrics

The age- and sex-standardized mortality densities of all-cause and CVD per 1000 person-years among adults with total CVH scores at the intermediate and high levels were significantly lower than those with low level (Fig. 1). Participants who achieved a higher CVH score had a significantly lower cumulative incidence rate of all-cause and CVD-specific mortality (*P* < 0.001 for all log-rank tests, Fig. 2).

During a median follow-up of 7.6 years (interquartile range: 4.3–10.9 years), compared with adults with a low total CVH score, those with intermediate or high scores had 40% (hazard ratio [HR] 0.60, 95% confidence interval [CI] 0.51–0.71) and 58% (HR 0.42, 95% CI 0.32–0.56) reduced risk of all-cause mortality after adjustment for all potential covariates (Table 2). Similar trends toward reduced risk of all-cause mortality were observed for higher individual CVH scores of diet, physical activity, nicotine, sleep health, and blood pressure (all *P* for trend < 0.05). However, the scores for BMI and blood lipids were not significantly associated with the risk of all-cause mortality. The adjusted PAF of high (score ≥ 75 points) vs. low or intermediate (score < 75 points) total CVH score with all-cause mortality was 33.4%, with the individual metric scores ranging from 4.7% for blood glucose to 16.5% for physical activity; when a score of ≥ 85 points was used to define “high score,” the adjusted PAF was 33.6%, with the individual metric scores ranging from 6.1 to 25.2% (Table 2).

**Table 2:** Adjusted hazard ratios of *Life’s Essential 8* cardiovascular health (CVH) score with risk of all-cause mortality, NHANES 2005–2018

CVH components	CVH score, HR (95% CI)			*P* for trend^c^	PAF	
	**0–49**	**50–74**	**75–100**			
					**Score ≥ 75 vs. < 75 points**	**Score ≥ 85 vs. < 85 points**
**Total**						
Cases/participants	711/4810	936/11,257	158/3884			
Age-, sex-, and race/ethnicity-adjusted	1.00 (reference)	0.48 (0.41–0.57)	0.28 (0.21–0.36)	< 0.001		
Fully adjusted^a^	1.00 (reference)	0.60 (0.51–0.71)	0.42 (0.32–0.56)	< 0.001	33.4	33.6
**Diet**						
Cases/participants	869/8761	498/5426	438/5764			
Age-, sex-, and race/ethnicity-adjusted	1.00 (reference)	0.74 (0.63–0.86)	0.57 (0.48–0.68)	< 0.001		
Fully adjusted^b^	1.00 (reference)	0.89 (0.77–1.04)	0.81 (0.68–0.98)	0.025	11.0	25.2
**Physical activity**						
Cases/participants	1277/11,133	66/1008	462/7810			
Age-, sex-, and race/ethnicity-adjusted	1.00 (reference)	0.61 (0.44–0.85)	0.52 (0.43–0.62)	< 0.001		
Fully adjusted^b^	1.00 (reference)	0.79 (0.58–1.10)	0.72 (0.60–0.87)	< 0.001	16.5	21.5
**Nicotine exposure**						
Cases/participants	623/5213	87/691	1095/14,047			
Age-, sex-, and race/ethnicity-adjusted	1.00 (reference)	0.69 (0.52–0.93)	0.42 (0.37–0.48)	< 0.001		
Fully adjusted^b^	1.00 (reference)	0.85 (0.64–1.15)	0.61 (0.52–0.73)	< 0.001	14.7	18.3
**Sleep health**						
Cases/participants	402/3508	392/4420	1011/12,023			
Age-, sex-, and race/ethnicity-adjusted	1.00 (reference)	0.61 (0.49–0.75)	0.54 (0.45–0.65)	< 0.001		
Fully adjusted^b^	1.00 (reference)	0.80 (0.64–0.99)	0.76 (0.63–0.92)	< 0.001	5.4	6.1
**Body mass index**						
Cases/participants	757/8422	582/6681	466/4848			
Age-, sex-, and race/ethnicity-adjusted	1.00 (reference)	0.68 (0.57–0.80)	0.89 (0.76–1.05)	0.019		
Fully adjusted^b^	1.00 (reference)	0.86 (0.72–1.02)	1.09 (0.93–1.29)	0.019	NA^d^	NA^d^
**Blood lipids (non-HDL cholesterol)**						
Cases/participants	662/7557	308/4697	835/7697			
Age-, sex-, and race/ethnicity-adjusted	1.00 (reference)	0.91 (0.72–1.15)	1.13 (0.99–1.29)	0.045		
Fully adjusted^b^	1.00 (reference)	1.01 (0.80–1.28)	1.12 (0.98–1.29)	0.045	NA^d^	NA^d^
**Blood glucose**						
Cases/participants	594/3660	669/7811	542/8480			
Age-, sex-, and race/ethnicity-adjusted	1.00 (reference)	0.56 (0.47–0.67)	0.51 (0.41–0.63)	< 0.001		
Fully adjusted^b^	1.00 (reference)	0.66 (0.55–0.81)	0.68 (0.55–0.84)	< 0.001	4.7	7.3
**Blood pressure**						
Cases/participants	779/5583	342/4051	684/10,317			
Age-, sex-, and race/ethnicity-adjusted	1.00 (reference)	0.77 (0.64–0.93)	0.76 (0.64–0.89)	0.002		
Fully adjusted ^b^	1.00 (reference)	0.85 (0.71–1.02)	0.83 (0.72–0.95)	0.002	5.8	18.2

*CVH* cardiovascular health, *CI* confidence interval, *HR* hazard ratio, *NA* not available, *PAF* population-attributable fraction

^a^Adjusted for sex, age, race/ethnicity, education level, marital status, ratio of family income poverty, and history of heart disease and stroke

^b^Adjusted for sex, age, race/ethnicity, education level, marital status, ratio of family income poverty, history of heart disease and stroke, diet, nicotine exposure, physical activity, sleep health, body mass index, blood glucose, blood lipids, and blood pressure. When the association between each CVH metric and mortality was evaluated, this metric was excluded from the adjustment

^c^Tests for linear trends across three categories of cardiovascular health metrics scores were performed by modeling the median value within each category as a continuous variable

^d^The individual PAFs were not calculated since the individual cardiovascular health metrics with adjusted HRs were ≥ 1.0 (e.g., BMI or blood lipids)

Compared with adults with a low total CVH score, those with intermediate and high scores were at 38% (HR 0.62, 95% CI 0.46–0.83) and 64% (HR 0.36, 95% CI 0.21–0.59) reduced risk of CVD-specific mortality after adjustment for all potential covariates. Similarly, higher scores of blood glucose were significantly associated with a reduced risk of CVD-specific mortality. Although there were non-significant associations for other individual CVH metrics including physical activity, sleep health, BMI, and blood pressure, similar trends were observed with higher scores associated with a reduced risk of CVD-specific mortality (all *P* for trend < 0.05). The adjusted PAF of high vs. low or intermediate total CVH score with CVD-specific mortality was 42.9%, with individual CVH metrics ranging from 2.2% for sleep health to 17.8% for physical activity; when a score of ≥ 85 points was used to define “high score,” the adjusted PAF was 70.6%, with the individual metric scores ranging from 2.2 to 16.2% (Table 3). The patterns of higher CVH scores in relation to reduced risk of all-cause and CVD-specific mortality remained consistent across subgroups of sex, age group, race/ethnicity, educational level, marital status, and family income to poverty (Additional file 1: Tables S2-S7), as well as after excluding adults with CVD history or death within the first 2 years of follow-up (Additional file 1: Table S8-S9).

**Table 3:** Adjusted hazard ratios of *Life’s Essential 8* cardiovascular health (CVH) score with risk of cardiovascular disease mortality, NHANES 2005–2018

CVH components	CVH score, HR (95% CI)			*P* for trend^c^	PAF	
	**0–49**	**50–74**	**75–100**			
					**Score ≥ 75 vs. < 75 points**	**Score ≥ 85 vs. < 85 points**
**Total**						
Cases/participants	209/4810	250/11,257	39/3884			
Age-, sex-, and race/ethnicity-adjusted	1.00 (reference)	0.50 (0.37–0.66)	0.24 (0.15–0.40)	< 0.001		
Fully adjusted^a^	1.00 (reference)	0.62 (0.46–0.83)	0.36 (0.21–0.59)	< 0.001	42.9	70.6
**Diet**						
Cases/participants	245/8761	132/5426	121/5764			
Age-, sex-, and race/ethnicity-adjusted	1.00 (reference)	0.77 (0.56–1.05)	0.64 (0.46–0.89)	0.007		
Fully adjusted^b^	1.00 (reference)	0.87 (0.63–1.22)	0.87 (0.59–1.27)	0.449	5.9	5.0
**Physical activity**						
Cases/participants	359/11,133	22/1008	117/7810			
Age-, sex-, and race/ethnicity-adjusted	1.00 (reference)	1.05 (0.57–1.93)	0.52 (0.39–0.70)	< 0.001		
Fully adjusted^b^	1.00 (reference)	1.40 (0.79–2.49)	0.74 (0.53–1.03)	0.103	17.8	15.2
**Nicotine exposure**						
Cases/participants	155/5213	23/691	320/14,047			
Age-, sex-, and race/ethnicity-adjusted	1.00 (reference)	0.76 (0.38–1.53)	0.57 (0.43–0.77)	< 0.001		
Fully adjusted^b^	1.00 (reference)	0.87 (0.42–1.79)	0.78 (0.55–1.09)	0.102	7.1	8.8
**Sleep health**						
Cases/participants	111/3508	114/4420	273/12,023			
Age-, sex-, and race/ethnicity-adjusted	1.00 (reference)	0.80 (0.56–1.15)	0.71 (0.52–0.98)	0.047		
Fully adjusted^b^	1.00 (reference)	1.07 (0.74–1.54)	0.98 (0.71–1.34)	0.750	2.2	2.2
**Body mass index**						
Cases/participants	231/8422	166/6681	101/4848			
Age-, sex-, and race/ethnicity-adjusted	1.00 (reference)	0.57 (0.42–0.76)	0.56 (0.40–0.79)	< 0.001		
Fully adjusted^b^	1.00 (reference)	0.72 (0.53–0.99)	0.75 (0.52–1.09)	0.067	9.7	9.7
**Blood lipids (non-HDL cholesterol)**						
Cases/participants	193/7557	85/4697	220/7697			
Age-, sex-, and race/ethnicity-adjusted	1.00 (reference)	0.90 (0.62–1.30)	0.96 (0.72–1.28)	0.836		
Fully adjusted^b^	1.00 (reference)	1.06 (0.73–1.54)	0.92 (0.69–1.22)	0.506	6.5	3.3
**Blood glucose**						
Cases/participants	186/3660	178/7811	134/8480			
Age-, sex-, and race/ethnicity-adjusted	1.00 (reference)	0.52 (0.38–0.70)	0.41 (0.29–0.57)	< 0.001		
Fully adjusted^b^	1.00 (reference)	0.68 (0.48–0.95)	0.63 (0.44–0.89)	0.031	10.3	10.2
**Blood pressure**						
Cases/participants	235/5583	100/4051	163/10,317			
Age-, sex-, and race/ethnicity-adjusted	1.00 (reference)	0.80 (0.57–1.13)	0.63 (0.46–0.87)	0.006		
Fully adjusted^b^	1.00 (reference)	0.94 (0.67–1.32)	0.74 (0.54–1.01)	0.047	12.5	16.2

*CVH* cardiovascular health, *CI* confidence interval, *HR* hazard ratio, *NA* not available, *PAF* population-attributable fraction

^a^Adjusted for sex, age, race/ethnicity, education level, marital status, ratio of family income poverty, and history of heart disease and stroke

^b^Adjusted for sex, age, race/ethnicity, education level, marital status, ratio of family income poverty, history of heart disease and stroke, diet, nicotine exposure, physical activity, sleep health, body mass index, blood glucose, blood lipids, and blood pressure. When the association between each CVH metric and mortality was evaluated, this metric was excluded from the adjustment

^c^Tests for linear trends across three categories of cardiovascular health metrics scores were performed by modeling the median value within each category as a continuous variable

^d^The individual PAFs were not calculated since the individual cardiovascular health metrics with adjusted HRs were ≥ 1.0

**Fig. 3:**
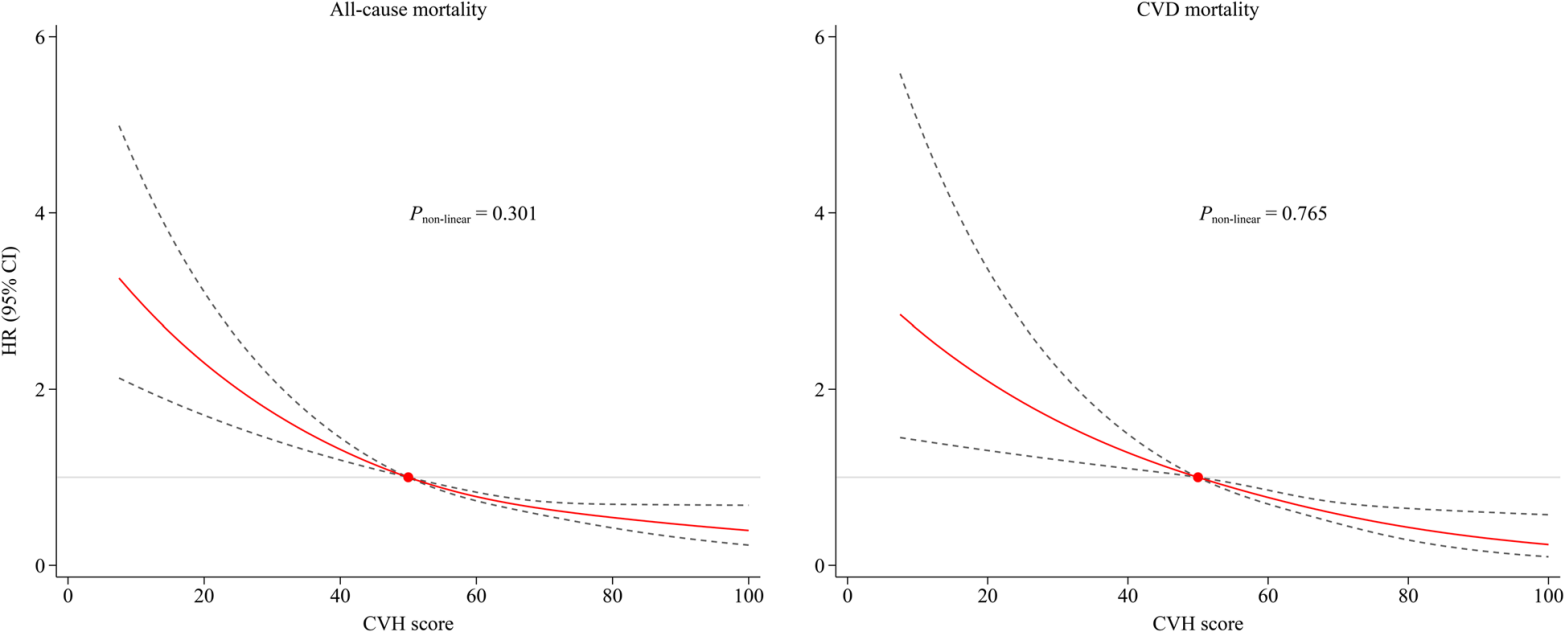
Dose–response associations of cardiovascular metric scores with all-cause and CVD-specific mortality. The reference is set at 50 points for the cardiovascular health score

There were approximately linear dose–response associations of total CVH scores with all-cause and CVD-specific mortality (all* P* for non-linear association > 0.05, Fig. 3). That is the risk of all-cause and CVD-specific mortality decreased linearly as total CVH scores increased.

## Discussion

Based on a large nationally representative sample of US adults, we found that adults with a high level of total or individual CVH metrics in *Life’s Essential 8* had a reduced risk of all-cause and CVD-specific mortality. There were approximately linear dose–response associations of increased total CVH metric score with reduced risk of all-cause and CVD-specific mortality. Our adjusted PAF estimates suggested that 33.4–33.5% of all-cause mortality and 42.9–70.6% of CVD mortality risk associated with low or intermediate total CVH could be avoided/eliminated if all of those were able to obtain a high CVH metric (depending on whether the score of ≥ 75 points or ≥ 85 points was used for “high score” definition). Our findings lend support to the new CVH metrics as part of *Life’s Essential 8* to have utility in the prediction of future all-cause and CVD-specific mortality.

Evidence to support a protective effect of ideal CVH in *Life’s Simple 7* on the development of diabetes, CVD, quality of life, and mortality later in life has been shown previously [7, 8, 13–27]. A meta-analysis of 13 cohort studies including 193,126 adults identified a linear dose–response association with a 1-point increase in ideal CVH metrics to associate with substantially reduced risk of all-cause (risk ratio [RR] = 0.89, 95%CI = 0.86–0.93) and CVD-specific (RR = 0.81, 95%CI = 0.76–0.86) mortality [8]. However, the included studies used a crude additive scoring method in *Life’s Simple 7* recommended by AHA in 2010, which may oversimplify the association between CVH metrics and mortality risk. For example, two individuals with largely different amounts of physical activity (e.g., one individual with 1 min/week vs. the other individual with 149 min/week) would both be classified as having intermediate physical activity. However, the dose–response analysis showed that the associations between these two doses of physical activity and mortality risk were substantially different [28, 29].

Based on an enhanced scoring algorithm ranging from 0 to 100 recommended in *Life’s Essential 8*, we found that a higher total CVH metric score was associated with a largely reduced risk of all-cause and CVD-specific mortality in a dose–response manner. Our findings suggest that a small improvement in total CVH score could lead to a large reduction in mortality. This is a very important finding of the present study. Particularly among those who might now have CVD or other chronic diseases (maybe some of them are unable to achieve a high CVH score because of functional or other limitations), the improvements in CVH score will still improve their risk relative. So, the improvement at any level of the CVH score has a benefit based on the spline plots, and those with the lower CVH score seem to benefit more from a small improvement. In all, the linear dose–response finding of our study suggests any improvement matters and especially for those with very low CVH. That is some improvements are better than none.

For the individual health behaviors, we found that physical inactivity was the major individual contributor to both all-cause and CVD-specific mortality. The association of adequate physical activity substantially reducing the risk of subsequent mortality is not new [13, 20], but our data validate the updated scoring of physical activity in *Life’s Essential 8* and underscore its key role in reducing the risk of mortality. We also found that nicotine exposure was the second important individual contributor to all-cause morality. Burnt cigarette and e-cigarette use is a serious public health issue among US adults and youth [30, 31], with data from cohort studies showing that no smoking (vs. current smoking) was associated with a reduced risk of all-cause mortality [13, 20]. We confirmed this association albeit based on the new definition of *Life’s Essential 8* by the AHA where nicotine exposure includes the use of inhaled nicotine delivery system and secondhand smoke exposure [9].

In addition, we found that an unhealthy diet was the third important individual contributor to all-cause mortality. There is much strong evidence supporting the benefits of healthy diets in the reduction of mortality risk [32, 33]. An important change in *Life’s Essential 8* was the assessment of diet. In *Life’s Simple 7*, the AHA recommended the use of only 5 aspects of diet to assess the dietary score, which included intakes of fruits and vegetables, fish, fiber-rich whole grains, sodium, and sugar-sweetened beverages [5]. However, in *Life’s Essential 8*, the AHA proposed a new, more comprehensive method for the assessment of dietary quality that incorporates the use of DASH-style eating patterns or the Healthy Eating Index 2015 for population-level assessment and Mediterranean-style eating patterns for individual assessment (especially in clinical settings). In this study, we used the Healthy Eating Index 2015 to assess dietary quality which was linked to mortality outcomes. In 2019, a national cohort study based on continuous NHANES surveys from 1999 to 2016 at baseline linked to 2011 mortality data showed that a diet score ≥ 69.3 using the Healthy Eating Index 2010 was associated with a reduced risk of all-cause mortality [20]. Our findings reinforce the above finding that a high dietary score defined by the Healthy Eating Index 2015 as a new metric with a broader scope of dietary types recommended by AHA was associated with a reduced risk of all-cause mortality. Overall, lifestyle modifications including evidence-based, effective, and appropriate public education and strategies to further promote a healthy diet are important to optimize CVH and thereby prevent CVD-related mortality.

A large body of evidence has shown short (< 7 h) or long (> 8 h) habitual sleep duration associates with all-cause and CVD-specific mortality [34, 35]. As a result of this evidence, a notable change in *Life’s Essential 8* by the AHA was the inclusion of a metric for sleep [9] in addition to the original 7 CVH metrics in *Life’s Simple 7* [5]. Taking advantage of sleep health as one of the AHA CVH metrics, we found that the risk for all-cause mortality significantly reduced with the increase in sleep metric score. Although we observed no significant association with CVD-specific mortality, patterns suggestive of a benefit to these outcomes were observed. However, it should be noted that the adjusted PAFs of sleep health with all-cause and CVD-specific mortality were relatively small (< 10%), suggesting that this CVH metric might be not more important than expected.

Similar to the previous findings based on the blood pressure metric in *Life’s Simple 7* [13, 20], we also found that blood pressure was an important individual contributor to CVD-specific mortality. However, inconsistent with the previous finding that HbA1c contributed least to CVD-specific mortality [13], we found that blood glucose (i.e., both fasting blood glucose and HbA1c were considered in *Life’s Essential 8*) was one of the three leading contributors to CVD-specific mortality. The difference might be explained by the change in definition for the fasting blood glucose metric in *Life’s Simple 7*, which likely did not comprehensively reflect the glycemic status and contribution to risk, and the various reference groups were used (i.e., score < 50 points in our study vs. HbA1c ≥ 5.7% reported by Yang et al. [13]). Using the blood glucose definition revised for *Life’s Essential 8* that includes both fasting blood glucose and HbA1c [9], our findings underline the importance of improving blood glucose to better reflect risk.

Consistent with a meta-analysis of 13 prospective studies among 193,126 participants, which determined the association between the ideal BMI metric defined by *Life’s Simple 7* and the risk of cardiovascular events or mortality [8], we found that the BMI score, as defined in *Life’s Essential 8*, was also not associated with all-cause and CVD-specific mortality. We also found that blood lipid score was not associated with all-cause and CVD-specific mortality, which was also consistent with previous data [8, 20]. Indeed, the U-shaped association of BMI and total cholesterol with CVD-specific mortality has been well-documented [36, 37], which might explain the non-statistically significant association we observed in our study given the AHA defined the lowest level of BMI or non-HDL cholesterol as the highest score in *Life’s Essential 8*. These findings suggest that the AHA should rescore the ideal level of BMI or blood lipids in future updates.

To our knowledge, this is the first large-scale, nationally representative cohort study to examine the association of total and individual CVH metrics using *Life’s Essential 8* and mortality risk. In addition, we performed dose–response associations between overall CVH score and mortality risk and found that an increased CVH score significantly lowered the risk of mortality. However, several potential limitations warrant consideration. First, four lifestyle factors including dietary intake, physical activity, nicotine exposure, and sleep health were self-reported, which may lead to a recall bias. Second, despite multivariable adjustment, additional confounding by measured or unmeasured covariates might have influenced the observed associations. Third, we only used measures of 8 CVH metrics in a single visit, and we did not consider metric trajectories or changes over time that might influence the observed association. However, previous studies have shown that CVH scores seem to be relatively stable or decline [38]. The potential for misclassification over time would be expected to bias the association toward the null because it is more likely that the change would occur randomly with respect to mortality. Fourth, our findings among US adults might not be directly generalized to other populations. Fifth, the sample size for cases of CVD-specific mortality in the categories of CVH scores was low in some instances (ranging from 22 to 359), which might explain the non-statistically significant associations of several individual CVH metrics with CVD-specific mortality we observed. Sixth, the overall CVH score in *Life’s Essential 8* is calculated using equal weights for each of the 8 CVH metrics. Seventh, the generalizability of our results to the whole US population should be made with caution due to the differences in several basic characteristics between follow-up participants and those without CVH metrics.

## Conclusions

We found that the prevalence of a high CVH score among US adults was low, and the total CVH score was inversely associated with all-cause and CVD-specific mortality. Physical inactivity, nicotine exposure, and unhealthy diet were the three leading individual contributors to all-cause mortality, whereas physical inactivity, elevated blood pressure, and elevated blood glucose were the three important ones to CVD-specific mortality. Our findings emphasize that primordial and primary prevention efforts on promoting CVH metrics, especially aiming at health factors and behaviors with high PAFs, should be strengthened to reduce early mortality risk later in life.

## Supplementary Information

**Additional file 1:** Supplementary results.** Fig S1.** Flow chart of inclusion/exclusion of participants. **Table S1.** Comparisons in differences of basic characteristic between participants with complete data and participants without CVH metrics. **Table S2.** Adjusted hazard ratios of all-cause and CVD-specific mortality by “Life’s Essential 8” cardiovascular health score and sex, NHANES 2005-2018. **Table S3.** Adjusted hazard ratios of all-cause and CVD-specific mortality by “Life’s Essential 8” cardiovascular health (CVH) score and age group, NHANES 2005-2018. **Table S4.** Adjusted hazard ratios of all-cause and CVD-specific mortality by “Life’s Essential 8” cardiovascular health (CVH) score and race/ethnicity, NHANES 2005-2018. **Table S5.** Adjusted hazard ratios of all-cause and CVD-specific mortality by “Life’s Essential 8” cardiovascular health (CVH) score and education level, NHANES 2005-2018. **Table S6.** Adjusted hazard ratios of all-cause and CVD-specific mortality by “Life’s Essential 8” cardiovascular health (CVH) score and marital status, NHANES 2005-2018. **Table S7.** Adjusted hazard ratios of all-cause and CVD-specific mortality by “Life’s Essential 8” cardiovascular health (CVH) score and ratio of family income to poverty, NHANES 2005-2018. **Table S8.** Adjusted hazard ratios of all-cause and CVD-specific mortality by “Life’s Essential 8” cardiovascular health (CVH) score after excluding adults with a history of CVD. **Table S9.** Adjusted hazard ratios of all-cause and CVD-specific mortality by “Life’s Essential 8” cardiovascular health (CVH) score after excluding death within the first 2 years of follow-up.

## Data Availability

Data from the National Health and Nutrition Examination Survey (NHANES) 2005–2018 are publicly available online (https://wwwn.cdc.gov/nchs/nhanes/Default.aspx).

## Abbreviations

AHA: American Heart Association
BMI: Body mass index
CI: Confidence interval
CVD: Cardiovascular disease
CVH: Cardiovascular health
FBG: Fasting blood glucose
HDL: High-density lipoprotein
HR: Hazard ratio
NHANES: National Health and Nutrition Examination Survey
PAF: Population-attributable fraction
RR: Risk ratio
US: United States
