# Supplementary material for: Association of the American Heart Association’s new “Life’s Essential 8” with all-cause and cardiovascular disease-specific mortality: prospective cohort study
Source: BMC Med. 2023 Mar 29;21:116. doi: 10.1186/s12916-023-02824-8 (PMC10053736; doi:10.1186/s12916-023-02824-8)
Supplement: Supplementary file 1 — Additional file 1: Supplementary results. Fig S1. Flow chart of inclusion/exclusion of participants. Table S1. Comparisons in differences of basic characteristic between participants with complete data and participants without CVH metrics. Table S2. Adjusted hazard ratios of all-cause and CVD-specific mortality by “Life’s Essential 8” cardiovascular health score and sex, NHANES 2005-2018. Table S3. Adjusted hazard ratios of all-cause and CVD-specific mortality by “Life’s Essential 8” cardiovascular health (CVH) score and age group, NHANES 2005-2018. Table S4. Adjusted hazard ratios of all-cause and CVD-specific mortality by “Life’s Essential 8” cardiovascular health (CVH) score and race/ethnicity, NHANES 2005-2018. Table S5. Adjusted hazard ratios of all-cause and CVD-specific mortality by “Life’s Essential 8” cardiovascular health (CVH) score and education level, NHANES 2005-2018. Table S6. Adjusted hazard ratios of all-cause and CVD-specific mortality by “Life’s Essential 8” cardiovascular health (CVH) score and marital status, NHANES 2005-2018. Table S7. Adjusted hazard ratios of all-cause and CVD-specific mortality by “Life’s Essential 8” cardiovascular health (CVH) score and ratio of family income to poverty, NHANES 2005-2018. Table S8. Adjusted hazard ratios of all-cause and CVD-specific mortality by “Life’s Essential 8” cardiovascular health (CVH) score after excluding adults with a history of CVD. Table S9. Adjusted hazard ratios of all-cause and CVD-specific mortality by “Life’s Essential 8” cardiovascular health (CVH) score after excluding death within the first 2 years of follow-up. [file 12916_2023_2824_MOESM1_ESM.doc]

**Fig S1 Flow chart of inclusion/exclusion of participants**

**Table S1.** **Comparisons in differences of basic characteristics of US Adults between participants with complete data and participants without CVH metrics**

| **Characteristic** | **Participants with complete data (n=19,951)** | **Participants without CVH metrics (n=8,208)** | ***p-*value** |
| --- | --- | --- | --- |
| **Sex (%)** |  |  | 0.016 |
| Male | 48.89 | 50.48 |  |
| Female | 51.11 | 49.52 |  |
| **Age group (%)** |  |  | < 0.001 |
| 30-49 y | 42.82 | 46.49 |  |
| 50-64 y | 33.90 | 31.16 |  |
| 65-79 y | 23.28 | 22.34 |  |
| **Race/Ethnicity (%)** |  |  | < 0.001 |
| Hispanic | 24.14 | 27.13 |  |
| Non-Hispanic White | 45.08 | 30.87 |  |
| Non-Hispanic Black | 21.07 | 25.23 |  |
| Other | 9.70 | 16.76 |  |
| **Education level (%)** |  |  | < 0.001 |
| <High school graduate | 22.74 | 32.88 |  |
| High school graduate | 22.74 | 21.98 |  |
| Some college or associates degree | 29.20 | 24.89 |  |
| College graduate or above | 25.32 | 20.25 |  |
| **Marital status (%)** |  |  | < 0.001 |
| Married | 59.02 | 53.60 |  |
| Divorced/separated/widowed | 23.71 | 25.98 |  |
| Unmarried/cohabitation | 17.27 | 20.42 |  |
| **Ratio of family income to poverty** |  |  | < 0.001 |
| <1.30 | 28.29 | 36.54 |  |
| 1.30-2.99 | 30.67 | 30.75 |  |
| ≥3.00 | 41.04 | 32.71 |  |
| **History of heart disease (%)** |  |  | 0.147 |
| Yes | 8.63 | 9.17 |  |
| No | 91.37 | 90.83 |  |
| **History of stroke (%)** |  |  | 0.002 |
| Yes | 3.81 | 4.60 |  |
| No | 96.19 | 95.4 |  |

**Table S2 Adjusted hazard ratios of all-cause and CVD-specific mortality by “Life's Essential 8” cardiovascular health score and sex, NHANES 2005-2018**

| **Subgroup** | **Total CVH score, HR (95% CI)** | | | ***P* for trend ^b^** |
| --- | --- | --- | --- | --- |
|  | **0-49** | **50-74** | **75-100** |  |
| **All-cause mortality** |  |  |  |  |
| **Male** |  |  |  |  |
| Cases/participants | 408/2,516 | 572/5,700 | 88/1,539 |  |
| Age-, sex- and race/ethnicity- adjusted | 1.00 (Reference) | 0.46 (0.37-0.58) | 0.28 (0.20-0.39) | < 0.001 |
| Fully adjusted ^a^ | 1.00 (Reference) | 0.54 (0.44-0.68) | 0.40 (0.27-0.59) | < 0.001 |
| **Female** |  |  |  |  |
| Cases/participants | 303/2,294 | 364/5,557 | 70/2,345 |  |
| Age-, sex- and race/ethnicity- adjusted | 1.00 (Reference) | 0.51 (0.40-0.65) | 0.28 (0.19-0.43) | < 0.001 |
| Fully adjusted ^a^ | 1.00 (Reference) | 0.71 (0.55-0.92) | 0.46 (0.29-0.71) | 0.001 |
| **CVD mortality** |  |  |  |  |
| **Male** |  |  |  |  |
| Cases/participants | 123/2,516 | 153/5,700 | 28/1,539 |  |
| Age-, sex- and race/ethnicity- adjusted | 1.00 (Reference) | 0.45 (0.31-0.65) | 0.30 (0.16-0.56) | < 0.001 |
| Fully adjusted ^a^ | 1.00 (Reference) | 0.52 (0.35-0.76) | 0.42 (0.22-0.81) | 0.001 |
| **Female** |  |  |  |  |
| Cases/participants | 86/2,294 | 97/5,557 | 11/2,345 |  |
| Age-, sex- and race/ethnicity- adjusted | 1.00 (Reference) | 0.59 (0.39-0.89) | 0.18 (0.08-0.39) | < 0.001 |
| Fully adjusted ^a^ | 1.00 (Reference) | 0.84 (0.55-1.29) | 0.28 (0.12-0.62) | 0.003 |

^a^ Adjusted for sex, age, race/ethnicity, education level, marital status, ratio of family income poverty, and history of heart disease and stroke.

^b^ Tests for linear trends across three categories of cardiovascular health metrics scores were performed by modeling the median value within each category as a continuous variable.

CVD, cardiovascular disease; CVH, cardiovascular health; CI, confidence interval; HR, hazard ratio

**Table S3. Adjusted hazard ratios of all-cause and CVD-specific mortality by “Life's Essential 8” cardiovascular health (CVH) score and age group, NHANES 2005-2018**

| **Subgroup** | **Total CVH score, HR (95% CI)** | | | ***P* for trend ^b^** |
| --- | --- | --- | --- | --- |
|  | **0-49** | **50-74** | **75-100** |  |
| **All-cause mortality** |  |  |  |  |
| **Age 30-49 years** |  |  |  |  |
| Cases/participants | 85/1,659 | 88/4,659 | 22/2,226 |  |
| Age-, sex- and race/ethnicity- adjusted | 1.00 (Reference) | 0.39 (0.26-0.57) | 0.22 (0.12-0.40) | < 0.001 |
| Fully adjusted ^a^ | 1.00 (Reference) | 0.53 (0.35-0.81) | 0.41 (0.20-0.83) | 0.006 |
| **Age 50-64 years** |  |  |  |  |
| Cases/participants | 264/1,928 | 241/3,832 | 35/1,003 |  |
| Age-, sex- and race/ethnicity- adjusted | 1.00 (Reference) | 0.35 (0.26-0.47) | 0.20 (0.11-0.34) | < 0.001 |
| Fully adjusted ^a^ | 1.00 (Reference) | 0.46 (0.34-0.63) | 0.32 (0.18-0.58) | < 0.001 |
| **Age 65-79 years** |  |  |  |  |
| Cases/participants | 362/1,223 | 607/2,766 | 101/655 |  |
| Age-, sex- and race/ethnicity- adjusted | 1.00 (Reference) | 0.64 (0.51-0.80) | 0.37 (0.27-0.51) | < 0.001 |
| Fully adjusted ^a^ | 1.00 (Reference) | 0.77 (0.62-0.95) | 0.51 (0.37-0.69) | < 0.001 |
| **CVD mortality** |  |  |  |  |
| **Age 30-49 years** |  |  |  |  |
| Cases/participants | 29/1,659 | 16/4,659 | 2/2,226 |  |
| Age-, sex- and race/ethnicity- adjusted | 1.00 (Reference) | 0.26 (0.11-0.59) | 0.07 (0.02-0.36) | < 0.001 |
| Fully adjusted ^a^ | 1.00 (Reference) | 0.40 (0.17-0.93) | 0.20 (0.04-1.17) | 0.015 |
| **Age 50-64 years** |  |  |  |  |
| Cases/participants | 68/1,928 | 47/3,832 | 6/1,003 |  |
| Age-, sex- and race/ethnicity- adjusted | 1.00 (Reference) | 0.27 (0.15-0.48) | 0.17 (0.05-0.56) | < 0.001 |
| Fully adjusted ^a^ | 1.00 (Reference) | 0.34 (0.19-0.63) | 0.26 (0.08-0.83) | 0.002 |
| **Age 65-79 years** |  |  |  |  |
| Cases/participants | 112/1,223 | 187/2,766 | 31/655 |  |
| Age-, sex- and race/ethnicity- adjusted | 1.00 (Reference) | 0.76 (0.55-1.05) | 0.37 (0.22-0.64) | < 0.001 |
| Fully adjusted ^a^ | 1.00 (Reference) | 0.89 (0.64-1.25) | 0.48 (0.27-0.85) | 0.011 |

^a^ Adjusted for sex, age, race/ethnicity, education level, marital status, ratio of family income poverty, and history of heart disease and stroke.

^b^ Tests for linear trends across three categories of cardiovascular health metrics scores were performed by modeling the median value within each category as a continuous variable.

CVD, cardiovascular disease; CVH, cardiovascular health; CI, confidence interval; HR, hazard ratio

**Table S4. Adjusted hazard ratios of all-cause and CVD-specific mortality by “Life's Essential 8” cardiovascular health (CVH) score and race/ethnicity, NHANES 2005-2018**

| **Subgroup** | **Total CVH score, HR (95% CI)** | | | ***P* for trend ^b^** |
| --- | --- | --- | --- | --- |
|  | **0-49** | **50-74** | **75-100** |  |
| **All-cause mortality** |  |  |  |  |
| **Hispanic** |  |  |  |  |
| Cases/participants | 102/1,053 | 137/2,979 | 23/785 |  |
| Age-, sex- and race/ethnicity- adjusted | 1.00 (Reference) | 0.58 (0.45-0.75) | 0.56 (0.36-0.88) | < 0.001 |
| Fully adjusted ^a^ | 1.00 (Reference) | 0.63 (0.49-0.83) | 0.65 (0.41-1.04) | 0.004 |
| **Non-Hispanic white** |  |  |  |  |
| Cases/participants | 378/2,151 | 562/4,854 | 110/1,989 |  |
| Age-, sex- and race/ethnicity- adjusted | 1.00 (Reference) | 0.54 (0.47-0.61) | 0.31 (0.25-0.39) | < 0.001 |
| Fully adjusted ^a^ | 1.00 (Reference) | 0.68 (0.60-0.78) | 0.52 (0.41-0.65) | < 0.001 |
| **Non-Hispanic black** |  |  |  |  |
| Cases/participants | 211/1,343 | 200/2,413 | 17/448 |  |
| Age-, sex- and race/ethnicity- adjusted | 1.00 (Reference) | 0.57 (0.47-0.70) | 0.35 (0.21-0.57) | < 0.001 |
| Fully adjusted ^a^ | 1.00 (Reference) | 0.70 (0.57-0.86) | 0.48 (0.29-0.80) | < 0.001 |
| **CVD mortality** |  |  |  |  |
| **Hispanic** |  |  |  |  |
| Cases/participants | 24/1,053 | 40/2,979 | 5/785 |  |
| Age-, sex- and race/ethnicity- adjusted | 1.00 (Reference) | 0.61 (0.32-1.17) | 0.92 (0.28-3.00) | 0.566 |
| Fully adjusted ^a^ | 1.00 (Reference) | 0.75 (0.39-1.48) | 1.37 (0.43-4.35) | 0.962 |
| **Non-Hispanic white** |  |  |  |  |
| Cases/participants | 87/1,469 | 148/4,854 | 27/1,989 |  |
| Age-, sex- and race/ethnicity- adjusted | 1.00 (Reference) | 0.52 (0.37-0.73) | 0.24 (0.14-0.42) | < 0.001 |
| Fully adjusted ^a^ | 1.00 (Reference) | 0.63 (0.44-0.90) | 0.34 (0.19-0.60) | < 0.001 |
| **Non-Hispanic black** |  |  |  |  |
| Cases/participants | 75/1,343 | 53/2,413 | 5/448 |  |
| Age-, sex- and race/ethnicity- adjusted | 1.00 (Reference) | 0.45 (0.29-0.70) | 0.24 (0.09-0.64) | < 0.001 |
| Fully adjusted ^a^ | 1.00 (Reference) | 0.65 (0.41-1.02) | 0.43 (0.16-1.13) | 0.024 |

^a^ Adjusted for sex, age, race/ethnicity, education level, marital status, ratio of family income poverty, and history of heart disease and stroke.

^b^ Tests for linear trends across three categories of cardiovascular health metrics scores were performed by modeling the median value within each category as a continuous variable.

CVD, cardiovascular disease; CVH, cardiovascular health; CI, confidence interval; HR, hazard ratio

**Table S5. Adjusted hazard ratios of all-cause and CVD-specific mortality by “Life's Essential 8” cardiovascular health (CVH) score and education level, NHANES 2005-2018**

| **Subgroup** | **Total CVH score, HR (95% CI)** | | | ***P* for trend ^b^** |
| --- | --- | --- | --- | --- |
|  | **0-49** | **50-74** | **75-100** |  |
| **All-cause mortality** |  |  |  |  |
| **<High school graduate** |  |  |  |  |
| Cases/participants | 303/1,548 | 276/2,598 | 24/391 |  |
| Age-, sex- and race/ethnicity- adjusted | 1.00 (Reference) | 0.48 (0.38-0.60) | 0.39 (0.21-0.70) | < 0.001 |
| Fully adjusted ^a^ | 1.00 (Reference) | 0.51 (0.40-0.65) | 0.41 (0.22-0.76) | < 0.001 |
| **High school graduate** |  |  |  |  |
| Cases/participants | 193/1,377 | 287/2,657 | 32/503 |  |
| Age-, sex- and race/ethnicity- adjusted | 1.00 (Reference) | 0.66 (0.51-0.84) | 0.44 (0.27-0.74) | < 0.001 |
| Fully adjusted ^a^ | 1.00 (Reference) | 0.81 (0.61-1.08) | 0.57 (0.34-0.96) | 0.033 |
| **Some college or AA degree** |  |  |  |  |
| Cases/participants | 171/1,395 | 226/3,462 | 40/968 |  |
| Age-, sex- and race/ethnicity- adjusted | 1.00 (Reference) | 0.38 (0.27-0.54) | 0.24 (0.14-0.41) | < 0.001 |
| Fully adjusted ^a^ | 1.00 (Reference) | 0.43 (0.30-0.62) | 0.31 (0.19-0.53) | < 0.001 |
| **College graduate or above** |  |  |  |  |
| Cases/participants | 44/490 | 147/2,540 | 62/2,022 |  |
| Age-, sex- and race/ethnicity- adjusted | 1.00 (Reference) | 0.85 (0.48-1.51) | 0.53 (0.29-0.98) | 0.010 |
| Fully adjusted ^a^ | 1.00 (Reference) | 0.99 (0.56-1.76) | 0.65 (0.34-1.25) | 0.069 |
| **CVD mortality** |  |  |  |  |
| **<High school graduate** |  |  |  |  |
| Cases/participants | 90/1,548 | 76/2,598 | 4/391 |  |
| Age-, sex- and race/ethnicity- adjusted | 1.00 (Reference) | 0.51 (0.33-0.78) | 0.21 (0.06-0.73) | < 0.001 |
| Fully adjusted ^a^ | 1.00 (Reference) | 0.53 (0.34-0.83) | 0.23 (0.06-0.84) | 0.001 |
| **High school graduate** |  |  |  |  |
| Cases/participants | 58/1,377 | 74/2,657 | 7/503 |  |
| Age-, sex- and race/ethnicity- adjusted | 1.00 (Reference) | 0.48 (0.29-0.79) | 0.32 (0.10-1.03) | 0.005 |
| Fully adjusted ^a^ | 1.00 (Reference) | 0.58 (0.33-1.02) | 0.39 (0.12-1.24) | 0.040 |
| **Some college or AA degree** |  |  |  |  |
| Cases/participants | 51/1,395 | 60/3,462 | 11/968 |  |
| Age-, sex- and race/ethnicity- adjusted | 1.00 (Reference) | 0.42 (0.24-0.75) | 0.25 (0.11-0.56) | < 0.001 |
| Fully adjusted ^a^ | 1.00 (Reference) | 0.54 (0.29-1.03) | 0.38 (0.15-0.96) | 0.032 |
| **College graduate or above** |  |  |  |  |
| Cases/participants | 10/490 | 40/2,540 | 17/2,022 |  |
| Age-, sex- and race/ethnicity- adjusted | 1.00 (Reference) | 1.67 (0.69-4.00) | 0.77 (0.28-2.18) | 0.141 |
| Fully adjusted ^a^ | 1.00 (Reference) | 1.83 (0.77-4.36) | 0.89 (0.32-2.46) | 0.248 |

^a^ Adjusted for sex, age, race/ethnicity, education level, marital status, ratio of family income poverty, and history of heart disease and stroke.

^b^ Tests for linear trends across three categories of cardiovascular health metrics scores were performed by modeling the median value within each category as a continuous variable.

CVD, cardiovascular disease; CVH, cardiovascular health; CI, confidence interval; HR, hazard ratio

**Table S6. Adjusted hazard ratios of all-cause and CVD-specific mortality by “Life's Essential 8” cardiovascular health (CVH) score and marital status, NHANES 2005-2018**

| **Subgroup** | **Total CVH score, HR (95% CI)** | | | ***P* for trend ^b^** |
| --- | --- | --- | --- | --- |
|  | **0-49** | **50-74** | **75-100** |  |
| **All-cause mortality** |  |  |  |  |
| **Married** |  |  |  |  |
| Cases/participants | 315/2,407 | 483/6,675 | 97/2,693 |  |
| Age-, sex- and race/ethnicity- adjusted | 1.00 (Reference) | 0.47 (0.38-0.60) | 0.31 (0.22-0.43) | < 0.001 |
| Fully adjusted ^a^ | 1.00 (Reference) | 0.57 (0.45-0.73) | 0.45 (0.32-0.63) | < 0.001 |
| **Divorced/separated/widowed** |  |  |  |  |
| Cases/participants | 298/1,469 | 328/2,657 | 39/605 |  |
| Age-, sex- and race/ethnicity- adjusted | 1.00 (Reference) | 0.53 (0.40-0.71) | 0.23 (0.14-0.39) | < 0.001 |
| Fully adjusted ^a^ | 1.00 (Reference) | 0.62 (0.46-0.84) | 0.31 (0.18-0.53) | < 0.001 |
| **Never married/cohabitation** |  |  |  |  |
| Cases/participants | 98/934 | 125/1,925 | 22/586 |  |
| Age-, sex- and race/ethnicity- adjusted | 1.00 (Reference) | 0.55 (0.37-0.82) | 0.40 (0.22-0.75) | 0.002 |
| Fully adjusted ^a^ | 1.00 (Reference) | 0.64 (0.43-0.96) | 0.57 (0.28-1.14) | 0.048 |
| **CVD mortality** |  |  |  |  |
| **Married** |  |  |  |  |
| Cases/participants | 89/2,407 | 128/6,675 | 23/2,693 |  |
| Age-, sex- and race/ethnicity- adjusted | 1.00 (Reference) | 0.41 (0.27-0.62) | 0.24 (0.12-0.48) | < 0.001 |
| Fully adjusted ^a^ | 1.00 (Reference) | 0.47 (0.31-0.71) | 0.33 (0.16-0.66) | < 0.001 |
| **Divorced/separated/widowed** |  |  |  |  |
| Cases/participants | 87/1,469 | 94/2,657 | 11/605 |  |
| Age-, sex- and race/ethnicity- adjusted | 1.00 (Reference) | 0.70 (0.46-1.06) | 0.24 (0.11-0.53) | < 0.001 |
| Fully adjusted ^a^ | 1.00 (Reference) | 0.84 (0.56-1.27) | 0.32 (0.14-0.74) | 0.012 |
| **Never married/cohabitation** |  |  |  |  |
| Cases/participants | 33/934 | 28/1,925 | 5/586 |  |
| Age-, sex- and race/ethnicity- adjusted | 1.00 (Reference) | 0.55 (0.27-1.13) | 0.36 (0.11-1.17) | 0.050 |
| Fully adjusted ^a^ | 1.00 (Reference) | 0.70 (0.35-1.39) | 0.57 (0.16-2.06) | 0.275 |

^a^ Adjusted for sex, age, race/ethnicity, education level, marital status, ratio of family income poverty, and history of heart disease and stroke.

^b^ Tests for linear trends across three categories of cardiovascular health metrics scores were performed by modeling the median value within each category as a continuous variable.

CVD, cardiovascular disease; CVH, cardiovascular health; CI, confidence interval; HR, hazard ratio

**Table S7. Adjusted hazard ratios of all-cause and CVD-specific mortality by “Life's Essential 8” cardiovascular health (CVH) score and ratio of family income to poverty, NHANES 2005-2018**

| **Subgroup** | **Total CVH score, HR (95% CI)** | | | ***P* for trend ^b^** |
| --- | --- | --- | --- | --- |
|  | **0-49** | **50-74** | **75-100** |  |
| **All-cause mortality** |  |  |  |  |
| **<1.30** |  |  |  |  |
| Cases/participants | 337/1,987 | 313/3,079 | 35/578 |  |
| Age-, sex- and race/ethnicity- adjusted | 1.00 (Reference) | 0.61 (0.49-0.76) | 0.62 (0.39-1.00) | 0.001 |
| Fully adjusted ^a^ | 1.00 (Reference) | 0.66 (0.53-0.84) | 0.70 (0.42-1.18) | 0.009 |
| **1.30-2.99** |  |  |  |  |
| Cases/participants | 247/1,604 | 360/3,580 | 48/935 |  |
| Age-, sex- and race/ethnicity- adjusted | 1.00 (Reference) | 0.62 (0.46-0.84) | 0.33 (0.21-0.53) | < 0.001 |
| Fully adjusted ^a^ | 1.00 (Reference) | 0.68 (0.50-0.92) | 0.38 (0.24-0.60) | < 0.001 |
| **≥3.00** |  |  |  |  |
| Cases/participants | 127/1,219 | 263/4,598 | 75/2,371 |  |
| Age-, sex- and race/ethnicity- adjusted | 1.00 (Reference) | 0.44 (0.31-0.62) | 0.29 (0.19-0.44) | < 0.001 |
| Fully adjusted ^a^ | 1.00 (Reference) | 0.48 (0.33-0.68) | 0.35 (0.23-0.53) | < 0.001 |
| **CVD mortality** |  |  |  |  |
| **<1.30** |  |  |  |  |
| Cases/participants | 93/1,987 | 79/3,079 | 8/578 |  |
| Age-, sex- and race/ethnicity- adjusted | 1.00 (Reference) | 0.55 (0.35-0.85) | 0.46 (0.19-1.13) | 0.010 |
| Fully adjusted ^a^ | 1.00 (Reference) | 0.60 (0.37-0.97) | 0.57 (0.23-1.39) | 0.054 |
| **1.30-2.99** |  |  |  |  |
| Cases/participants | 87/1,469 | 100/3,580 | 14/935 |  |
| Age-, sex- and race/ethnicity- adjusted | 1.00 (Reference) | 0.64 (0.42-0.98) | 0.27 (0.14-0.54) | < 0.001 |
| Fully adjusted ^a^ | 1.00 (Reference) | 0.71 (0.47-1.07) | 0.30 (0.14-0.65) | 0.001 |
| **≥3.00** |  |  |  |  |
| Cases/participants | 35/1,219 | 71/4,598 | 17/2,371 |  |
| Age-, sex- and race/ethnicity- adjusted | 1.00 (Reference) | 0.49 (0.27-0.92) | 0.27 (0.12-0.64) | 0.003 |
| Fully adjusted ^a^ | 1.00 (Reference) | 0.53 (0.29-0.97) | 0.33 (0.14-0.77) | 0.009 |

^a^ Adjusted for sex, age, race/ethnicity, education level, marital status, ratio of family income poverty, and history of heart disease and stroke.

^b^ Tests for linear trends across three categories of cardiovascular health metrics scores were performed by modeling the median value within each category as a continuous variable.

CVD, cardiovascular disease; CVH, cardiovascular health; CI, confidence interval; HR, hazard ratio

**Table S8. Adjusted hazard ratios of all-cause and CVD-specific mortality by “Life's Essential 8” cardiovascular health (CVH) score after excluding adults with a history of CVD**

| **Subgroup** | **Total CVH score, HR (95% CI)** | | | ***P* for trend ^b^** |
| --- | --- | --- | --- | --- |
|  | **0-49** | **50-74** | **75-100** |  |
| **All-cause mortality** |  |  |  |  |
| Cases/participants | 452/3,876 | 665/10,153 | 119/3,716 |  |
| Age-, sex- and race/ethnicity- adjusted | 1.00 (Reference) | 0.46 (0.38-0.55) | 0.27 (0.20-0.37) | < 0.001 |
| Fully adjusted ^a^ | 1.00 (Reference) | 0.54 (0.45-0.65) | 0.38 (0.27-0.52) | < 0.001 |
| **CVD mortality** |  |  |  |  |
| Cases/participants | 120/3,876 | 148/10,153 | 25/3,716 |  |
| Age-, sex- and race/ethnicity- adjusted | 1.00 (Reference) | 0.42 (0.29-0.61) | 0.18 (0.09-0.34) | < 0.001 |
| Fully adjusted ^a^ | 1.00 (Reference) | 0.48 (0.33-0.70) | 0.23 (0.12-0.43) | < 0.001 |

^a^ Adjusted for sex, age, race/ethnicity, education level, marital status, ratio of family income poverty, and history of heart disease and stroke.

^b^ Tests for linear trends across three categories of cardiovascular health metrics scores were performed by modeling the median value within each category as a continuous variable.

CVD, cardiovascular disease; CVH, cardiovascular health; CI, confidence interval; HR, hazard ratio

**Table S9. Adjusted hazard ratios of all-cause and CVD-specific mortality by “Life's Essential 8” cardiovascular health (CVH) score after excluding death within the first 2 years of follow-up**

| **Subgroup** | **Total CVH score, HR (95% CI)** | | | ***P* for trend ^b^** |
| --- | --- | --- | --- | --- |
|  | **0-49** | **50-74** | **75-100** |  |
| **All-cause mortality** |  |  |  |  |
| Cases/participants | 598/4,697 | 800/11,121 | 139/3,865 |  |
| Age-, sex- and race/ethnicity- adjusted | 1.00 (Reference) | 0.50 (0.42-0.60) | 0.28 (0.21-0.37) | < 0.001 |
| Fully adjusted ^a^ | 1.00 (Reference) | 0.62 (0.51-0.75) | 0.41 (0.30-0.55) | < 0.001 |
| **CVD mortality** |  |  |  |  |
| Cases/participants | 181/4,782 | 218/11,225 | 35/3,880 |  |
| Age-, sex- and race/ethnicity- adjusted | 1.00 (Reference) | 0.49 (0.37-0.66) | 0.21 (0.13-0.34) | < 0.001 |
| Fully adjusted ^a^ | 1.00 (Reference) | 0.62 (0.45-0.84) | 0.31 (0.18-0.52) | < 0.001 |

^a^ Adjusted for sex, age, race/ethnicity, education level, marital status, ratio of family income poverty, and history of heart disease and stroke.

^b^ Tests for linear trends across three categories of cardiovascular health metrics scores were performed by modeling the median value within each category as a continuous variable.

CVD, cardiovascular disease; CVH, cardiovascular health; CI, confidence interval; HR, hazard ratio
